# Efficacy and safety of pemafibrate administration in patients with dyslipidemia: a systematic review and meta-analysis

**DOI:** 10.1186/s12933-019-0845-x

**Published:** 2019-03-21

**Authors:** Satoshi Ida, Ryutaro Kaneko, Kazuya Murata

**Affiliations:** Department of Diabetes and Metabolism, Ise Red Cross Hospital, 1-471-2, Funae, 1-Chome, Ise-shi, Mie 516-8512 Japan

**Keywords:** Fibrate, Meta-analysis, Dyslipidemia, Pemafibrate, Adverse events, Lipid profile, Hepatobiliary enzyme activity

## Abstract

**Background:**

Using a meta-analysis of randomized controlled trials (RCTs), this study aimed to investigate the efficacy and safety of pemafibrate, a novel selective peroxisome proliferator-activated receptor α modulator, in patients with dyslipidemia.

**Methods:**

A search was performed using the MEDLINE, Cochrane Controlled Trials Registry, and ClinicalTrials.gov databases. We decided to employ RCTs to evaluate the effects of pemafibrate on lipid and glucose metabolism-related parameters in patients with dyslipidemia. For statistical analysis, standardized mean difference (SMD) or odds ratio (OR) and 95% confidence intervals (CIs) were calculated using the random effect model.

**Results:**

Our search yielded seven RCTs (with a total of 1623 patients) that satisfied the eligibility criteria of this study; hence, those studies were incorporated into this meta-analysis. The triglyceride concentration significantly decreased in the pemafibrate group (SMD, − 1.38; 95% CI, − 1.63 to − 1.12; P < 0.001) than in the placebo group, with a reduction effect similar to that exhibited by fenofibrate. Compared with the placebo group, the pemafibrate group also showed improvements in high-density and non-high-density lipoprotein cholesterol levels as well as in homeostasis model assessment for insulin resistance. Furthermore, the pemafibrate group showed a significant decrease in hepatobiliary enzyme activity compared with the placebo and fenofibrate groups; and, total adverse events (AEs) were significantly lower in the pemafibrate group than in the fenofibrate group (OR, 0.60; 95% CI, 0.49–0.73; P < 0.001). In contrast, the low-density lipoprotein cholesterol level was significantly higher in the pemafibrate group than in the placebo (P = 0.006) and fenofibrate (P < 0.001) groups.

**Conclusions:**

The lipid profile significantly improved in the pemafibrate group than in the placebo group. In addition to the pemafibrate group having an improved lipid profile, which was comparable with that of the fenofibrate group, the AEs were significantly lower than in the fenofibrate group and an improvement in hepatobiliary enzyme activity was also recognized. However, we believe that actual clinical data as well as long-term efficacy and safety need to be investigated in the future.

**Electronic supplementary material:**

The online version of this article (10.1186/s12933-019-0845-x) contains supplementary material, which is available to authorized users.

## Background

Previous studies have demonstrated that lipid control is important for preventing cardiovascular disease onset [1, 2]. In particular, the finding that cardiovascular disease onset can be prevented by reducing low-density lipoprotein cholesterol (LDL-C) levels via statin administration [1] has achieved high clinical priority. However, the problem here is that the benefit of statin administration to cardiovascular disease inhibition is only approximately 20%, the remaining 80% being residual risk [3]. Target lipids for arteriosclerosis prevention, including triglycerides (TG) and high-density lipoprotein cholesterol (HDL-C) except LDL-C, and hypertriglyceridemia and low HDL-C-emia are reportedly associated with cardiovascular disease onset [4]. As a drug against hyperTG-emia and low HDL-C-emia, a fibrate-system drug capable of strong TG-lowering and HDL-C-elevating actions is being clinically used to activate peroxisome proliferator-activated receptor α (PPARα) [5]. Some meta-analyses have reported the possibility of the suppression of cardiovascular disease development by fibrate administration [6, 7]; this may be useful in remedying the residual risk of arteriosclerosis that cannot be sufficiently controlled by statin administration alone. However, manifestation of side effects, such as hepatic disorder, kidney disorder, or rhabdomyolysis, may be problematic for fibrate-type drugs and may occasionally make it difficult to continue administration [8, 9]. Thus, fibrate formulations are presently considered problematic for wide-scale clinical use.

Pemafibrate reportedly reduces TG, which is considered as a risk factor for the development of cardiovascular diseases, or increases HDL-C levels. Furthermore, pemafibrate is a PPARα modulator (SPPARMα) with extremely high selectivity to PPARs subtypes, developed with the aim to reduce the occurrence of adverse effects associated with fibrate-type drugs and as a drug with fewer restrictions for use in patients with renal disease or for concomitant statin use [10]. Pemafibrate possesses lipid-improving effects similar to those of existing fibrate-type drugs; moreover, its side effects are equivalent to those of the placebo [11, 12]. We considered that highly relevant results related to the efficacy and safety of pemafibrate administration could be obtained via an integrated analysis of all these previous studies. Therefore, using a meta-analysis of randomized controlled trials (RCTs), this study aimed to investigate the efficacy and safety of pemafibrate administration in patients with dyslipidemia.

## Methods

### Study selection

The literature search was performed on November 1, 2018, using MEDLINE (from 1960), Cochrane Controlled Trials Registry (from 1960), and ClinicalTrials.gov. The search strategy included terms of “(pemafibrate or selective peroxisome proliferator-activated receptor or selective peroxisome proliferator-activated receptor or selective modulator of peroxisome proliferator-activated receptor or selective modulator of peroxisome proliferator-activated receptor or selective PPAR or selective modulator of PPAR) AND (Randomized Controlled Trial or Controlled Clinical Trial or Randomized or Randomized or placebo or randomly or assigned).” We decided to include RCTs to evaluate the effect of pemafibrate on lipid and glucose metabolism-related parameters in patients with dyslipidemia. The RCTs included compared pemafibrate with placebo and lipid-lowering drugs irrespective of the presence of diet/exercise therapy or the use of lipid-lowering drugs. Exclusion criteria included studies that were not RCT, research on animals, research without sufficient data to perform analysis, and cases of duplicate literature. In cases of difficulty in interpretation, the senior author decided to consult with other reviewers (RK and KM).

### Data extraction and quality assessment

We created a data extraction form describing the characteristics of the research included in each study (key author’s name, publication year, study location, sample size, patient’s baseline information, basic treatment, and treatment duration). Continuous variables were expressed as the mean value, standard deviation, standard error or 95% confidence intervals (CIs), and binary variables were expressed in terms of percentage (%). In case of a study comparing one placebo group and two or more intervention groups, we treated it as two or more studies sharing the placebo group. For quality evaluation, we used Cochrane’s risk of bias tool [13]. We evaluated low risk of bias, moderate risk of bias, and high risk of bias related to six domains (random sequence generation, allocation concealment, blinding of personnel and participants, blinding of outcome assessors, incomplete data, and selective reporting).

### Statistical analysis

As an indicator of the therapeutic effect, the difference between groups was assessed regarding the amount of change of lipid and sugar metabolism-related markers before and after treatment. Since studies used different units, we decided to analyze using standardized mean difference (SMD) and 95% CIs. As a safety evaluation index, analysis was performed using odds ratio (OR) and 95% CIs related to total AEs, the increase in hepatobiliary enzyme activities [aspartate aminotransferase (AST), alanine aminotransferase (ALT), and γ-glutamyl transpeptidase (γGTP) activities above the normal upper limit], kidney disorder (creatinine 1.5 mg/dL or more), and creatine kinase (CK) increase (above the normal upper limit). In cases where only the standard error or P-values were mentioned, the standard deviation was calculated with reference to Altman and Bland [14]. In the absence of the standard deviation, it was calculated from 95% CIs, t-values, or P-values [15]. A random effect model was used for the analysis, and I^2^ was used for the evaluation of the statistical heterogeneity; it was assumed that heterogeneity was observed in case of I^2^ ≥ 50% [16]. If the number of RCTs incorporated in the analysis was 10 or more, we would create a funnel plot by evaluation of the systematic review [15]. Regarding the comparative study of the effects of pemafibrate and placebo on TG, we decided to perform subgroup analysis including: (1) baseline TG ≥ 300 mg/dL and TG < 300 mg/dL type, (2) presence/absence of diabetes type and (3) presence/absence of concomitant statin use type. For analysis, RevMan version 5.3 (Cochrane Collaboration, http://tech.cochrane.org/revman/download, December/2018) was used.

## Results

### Description of studies included and assessment of potential bias

**Fig. 1:**
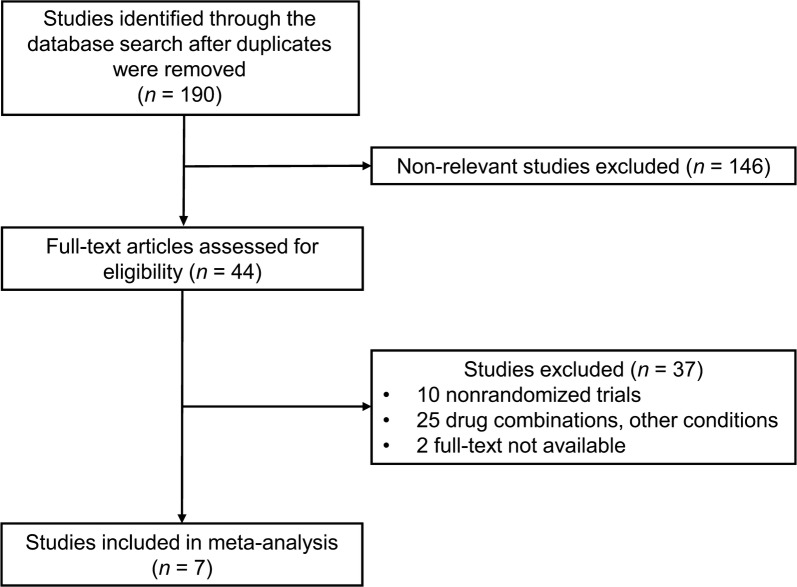
Study flow diagram

In total, 190 papers were extracted from the literature, from which seven RCTs (involving 1623 patients) satisfied the eligibility criteria of this study and were incorporated into the meta-analysis (Fig. 1) [11, 12, 17–21]. The characteristics of these seven RCTs are summarized in Table 1. The dose of pemafibrate in these studies was 0.025, 0.05, 0.1, 0.2, or 0.4 mg/day. The comparison group was placebo and fenofibrate (100, 106.6, or 200 mg/day). Mean patient age was 52 years, the mean ratio of female patients was 16.9%, and the mean examination period was 16 weeks.

**Table 1:** Characteristics of pemafibrate interventions included in the present meta-analysis compared with placebo or fenofibrate interventions

No.	References	Year	Region	No. of patients	Age (years)	% women	BMI (kg/m^2^)	Diabetes (%)	Hypertension (%)	Statin (%)	Study duration (weeks)	TG (mmol/L)
1	Ishibashi et al. [11]	2016	Japan	224	48	2.9	26.8	11.4	25.7	0	12	3.4
2	Arai et al. [17]	2017	Japan	423	55	20	26.2	29.3	58.5	100	24	4.3
3	Arai et al. [12]	2018	Japan	526	49	12	26.9	16.3	34.9	0	12	346 (mg/dL)
4	Araki et al. [18]	2018	Japan	166	61	34	26	100	37	40.4	24	3.2
5	Ishibashi et al. [19]	2018	Japan	225	53	21	26.1	6.8	24.7	0	24	2.7
6	Matsuba et al. [20]	2018	Japan	27	46	0	25.8	0	18.2	0	12	3.4
7	Yamashita et al. [21]	2018	Japan	32	52	29	25	12.5	31.3	0	4	2.9

Unless indicated otherwise, data are shown as mean values

*BMI* body mass index, *TG* triglycerides

In the RCTs included in this study, the proportion of appropriate assessment by each domain was 85.7% (6/7) for random sequence generation, 85.7% (6/7) for allocation concealment, 85.7% (6/7) for blinding of participants and personnel, 85.7% (6/7) for blinding of outcome assessors, 85.7% (6/7) for incomplete data and 100% (7/7) for selective reporting. Generally speaking, the quality of the RCTs included was high. As the number of RCTs included was less than 10, a funnel plot was not created (see Additional file 1: Table S1).

### Efficacy

**Fig. 2:**
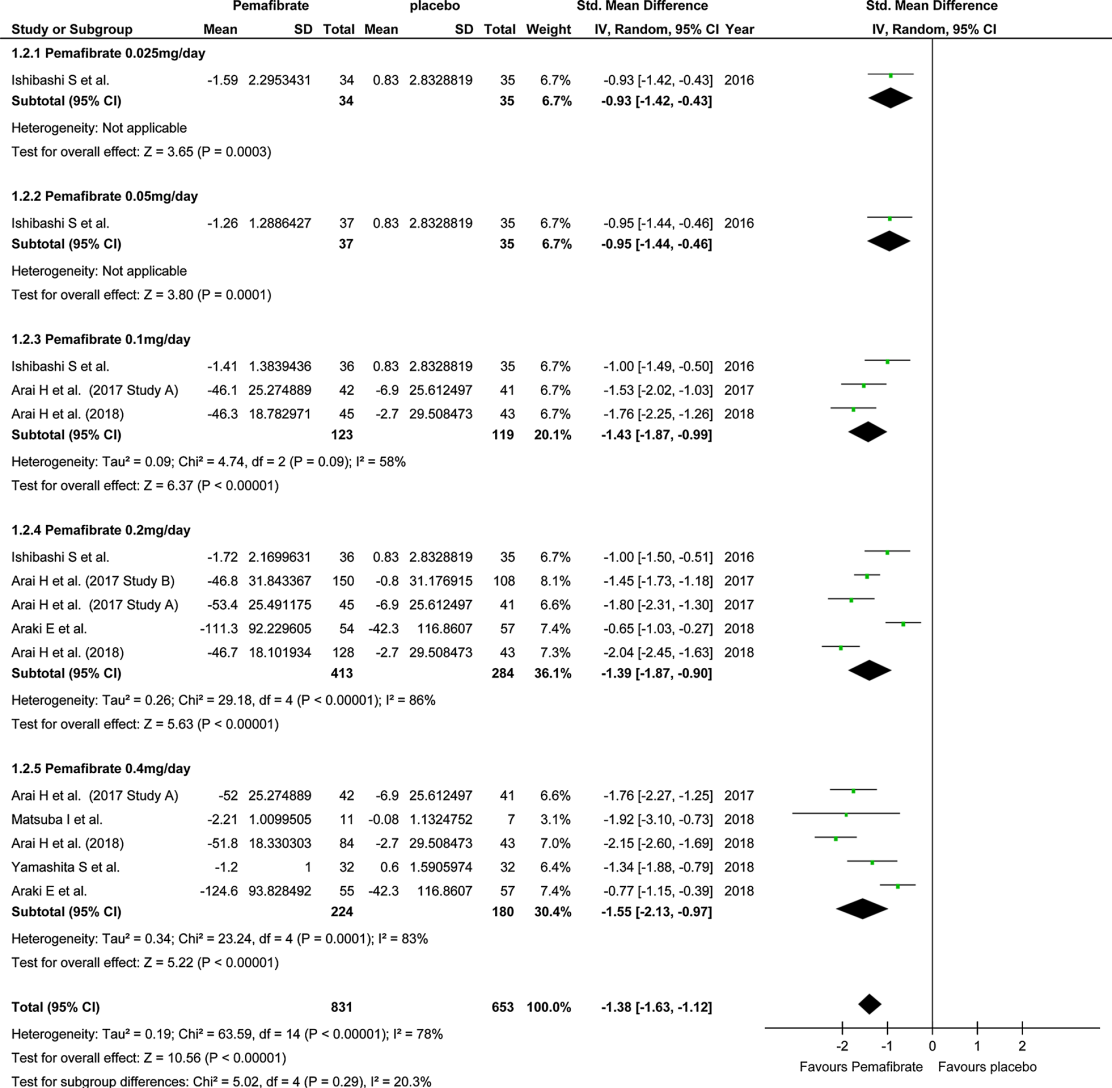
Forest plot presenting meta-analysis based on SMDs for the effect pemafibrate versus placebo on TGs. SMDs in the individual studies are presented as squares with 95% confidence intervals (CIs) presented as extending lines. The pooled SMD with its 95% CI is depicted as a diamond. SMDs, standardized mean differences; TG, triglycerides

**Fig. 3:**
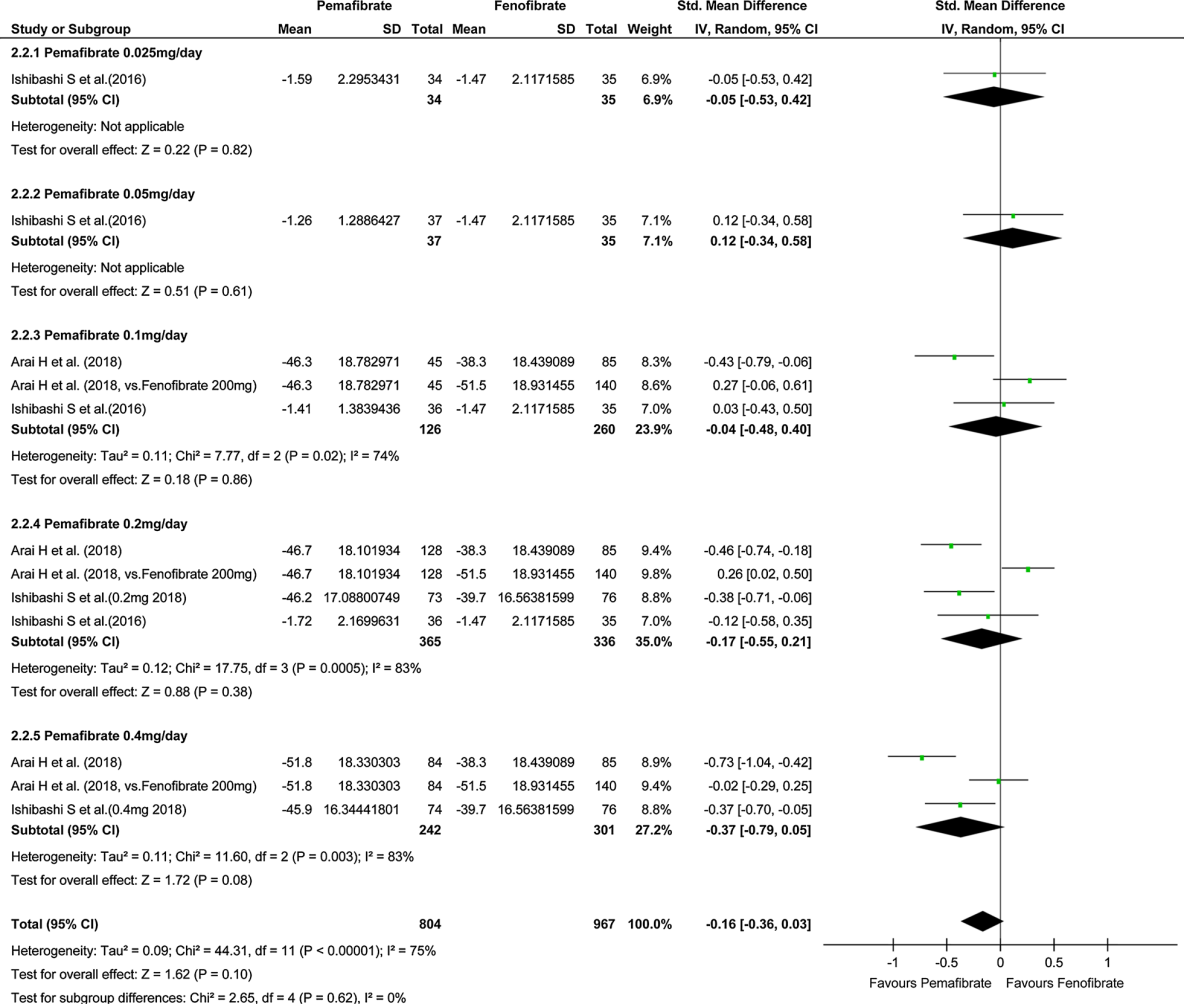
Forest plot presenting meta-analysis based on SMDs for effect of pemafibrate versus fenofibrate on TGs. SMDs in the individual studies are presented as squares with 95% confidence intervals (CIs) presented as extending lines. The pooled SMD with its 95% CI is depicted as a diamond. SMDs, standardized mean differences; TG, triglycerides

In the study on the effect on TG level, the number of pooled subjects was 831 in the pemafibrate group and 653 in the placebo group. The statistical heterogeneity was I^2^ = 78% (P < 0.001), indicating significant heterogeneity. In the pemafibrate group, TG level decreased significantly compared with the placebo group (SMD, − 1.38; 95% CI, − 1.63 to − 1.12; P < 0.001; Fig. 2). Regardless of the dose of pemafibrate, TG in the pemafibrate group decreased significantly compared to the placebo group. On the other hand, there was no significant difference in TG level between the pemafibrate and fenofibrate groups (SMD, − 0.16; 95% CI, − 0.36 to 0.03; P = 0.10; Fig. 3).

In the study on the influence on HDL-C level, the number of pooled subjects was 722 in the pemafibrate group and 539 in the placebo group. The statistical heterogeneity was I^2^ = 0% (P = 0.45), meaning that no heterogeneity was observed. In the pemafibrate group, HDL-C level increased significantly compared with the placebo group (SMD, 0.77; 95% CI, 0.66–0.89; P < 0.001) (see Additional file 1: Fig. S1). Regardless of the dose of pemafibrate, HDL-C level in the pemafibrate group decreased significantly compared to the placebo group. On the other hand, there was no significant difference in HDL-C level between the pemafibrate and fenofibrate groups.

In the study on the effect on LDL-C level, the number of pooled subjects was 711 in the pemafibrate group and 532 in the placebo group. The statistical heterogeneity was I^2^ = 28% (P = 0.17), meaning that no heterogeneity was observed. In the pemafibrate group, LDL-C level increased significantly, compared with the placebo group (SMD, 0.19; 95% CI, 0.06–0.33; P = 0.006) (see Additional file 1: Fig. S2). Furthermore, in the pemafibrate group, the LDL-C level was significantly higher than in the fenofibrate group, an effect which was particularly marked at the 0.4 mg dose pemafibrate group. In the study on the influence on non-HDL-C level, the number of pooled subjects was 722 in the pemafibrate group and 539 in the placebo group. The statistical heterogeneity was I^2^ = 0% (P = 0.58) and no heterogeneity was observed. The non-HDL-C level was significantly lower in the pemafibrate group than in the placebo group (SMD, − 0.39; 95% CI, − 0.51 to − 0.28; P < 0.001) (see Additional file 1: Fig. S3). On the other hand, no significant difference in non-HDL-C level was observed between the pemafibrate and fenofibrate groups.

In the study related to the influence on homeostasis model assessment for insulin resistance (HOMA-IR), the number of pooled subjects was 585 in the pemafibrate group and 507 in the placebo group. The statistical heterogeneity was I^2^ = 0% (P = 0.94), indicating no heterogeneity. In the pemafibrate group, the HOMA-IR was significantly lower than in the placebo group (SMD, − 0.27; 95% CI, − 0.39 to − 0.14; P < 0.001) (see Additional file 1: Fig. S4). On the other hand, there was no significant difference in HOMA-IR between the pemafibrate and fenofibrate groups. In the study on the influence on HbA1c level, the number of pooled subjects was 290 in the pemafibrate group and 238 in the placebo group. The statistical heterogeneity was I^2^ = 0% (P = 0.94), indicating no heterogeneity. There was no significant difference in HbA1c level between the pemafibrate group and the placebo group (SMD, 0.03; 95% CI, − 0.15 to 0.20; P = 0.76) (see Additional file 1: Fig. S5), while no significant difference was observed in HbA1c level between the pemafibrate group and the fenofibrate group.

According to the results of subgroup analysis on the effect on TG level in the pemafibrate and placebo groups, the TG level decreased significantly in the pemafibrate group compared with the placebo group, regardless of the baseline TG value, the presence or absence of diabetes and the presence or absence of the concomitant use of statin (see Additional file 1: Figs. S6–S8).

### Safety

**Fig. 4:**
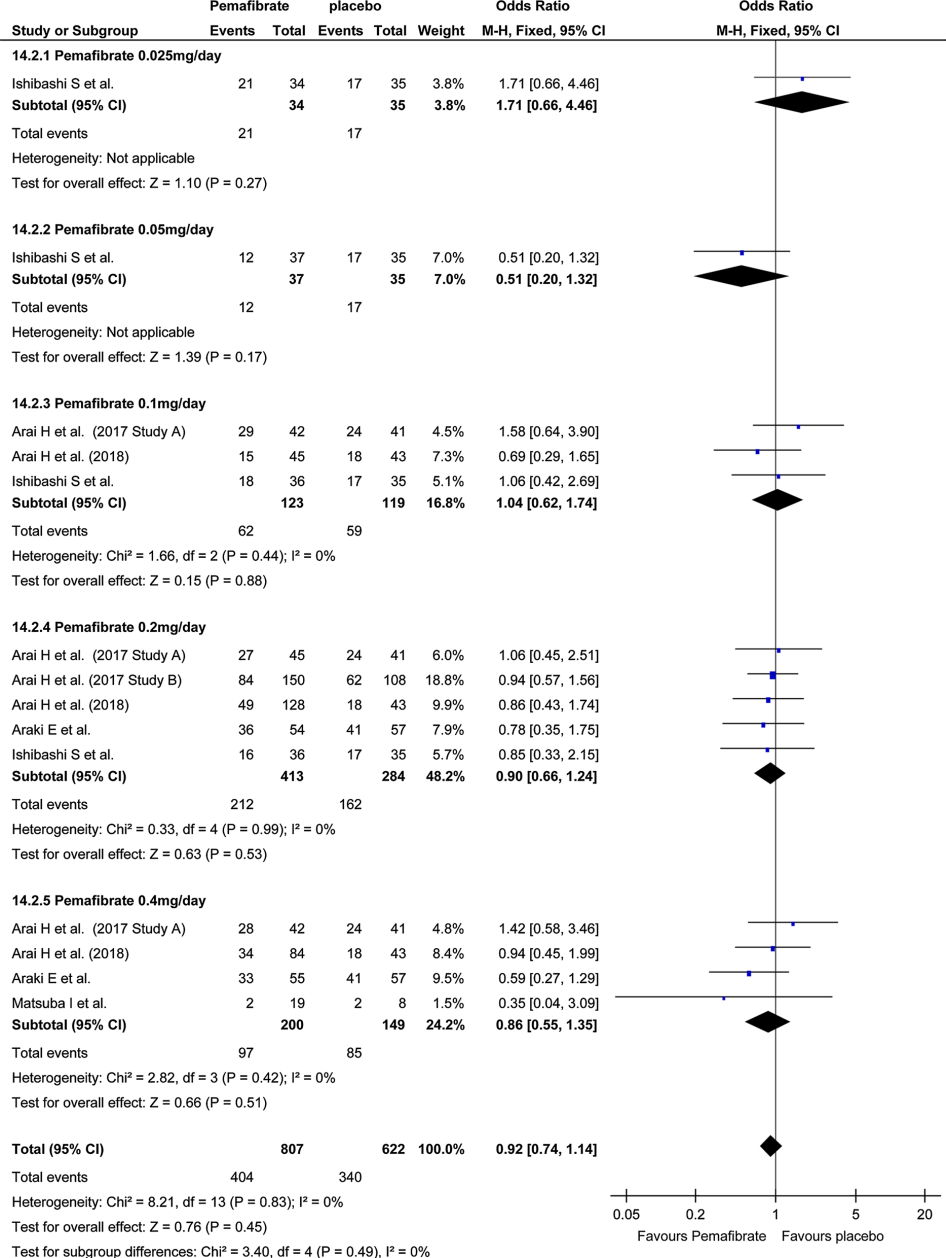
Forest plot presenting meta-analysis on OR for effect of pemafibrate versus placebo on total AEs. OR in the individual studies are presented as squares with 95% confidence intervals (CIs) presented as extending lines. The pooled OR with its 95% CI is depicted as a diamond. OR, odds ratio; AEs, adverse events

**Fig. 5:**
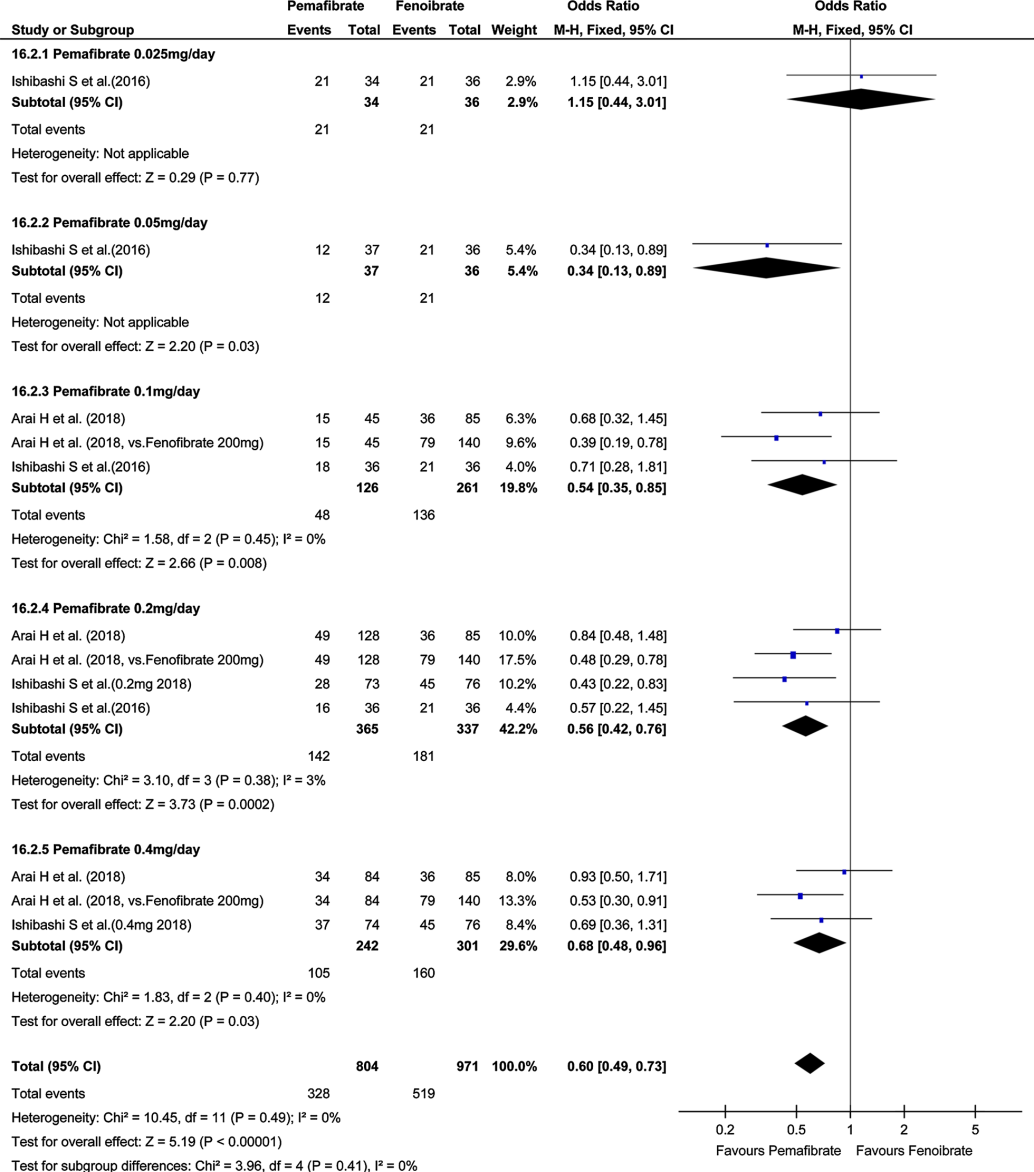
Forest plot presenting meta-analysis on OR for effect of pemafibrate versus fenofibrate on total AEs. OR in the individual studies are presented as squares with 95% confidence intervals (CIs) presented as extending lines. The pooled OR with its 95% CI is depicted as a diamond. OR, odds ratio; AEs, adverse events

**Fig. 6:**
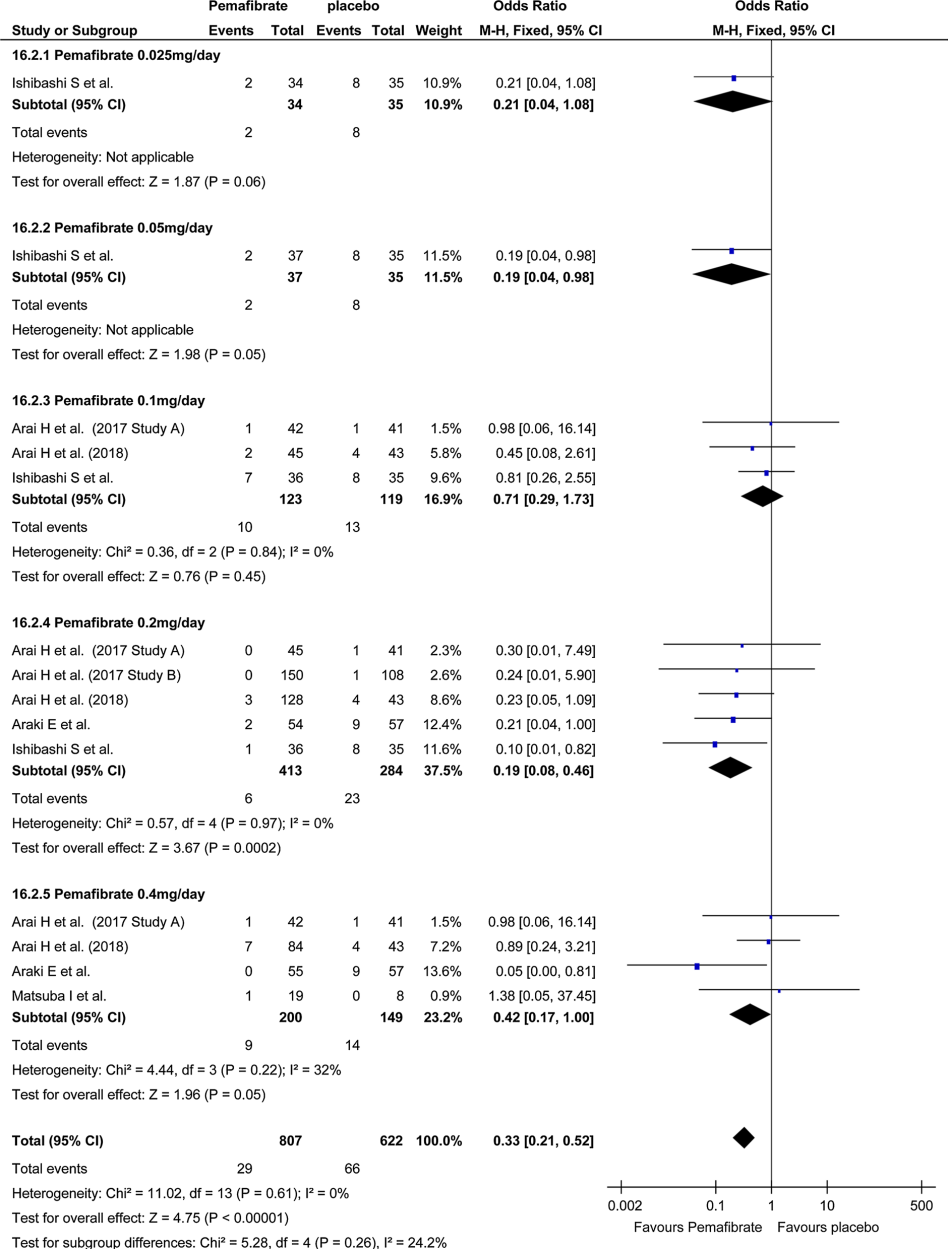
Forest plot presenting meta-analysis on OR for effect of pemafibrate versus placebo on hepatobiliary enzyme. OR in the individual studies are presented as squares with 95% confidence intervals (CIs) presented as extending lines. The pooled OR with its 95% CI is depicted as a diamond. OR, odds ratio

**Fig. 7:**
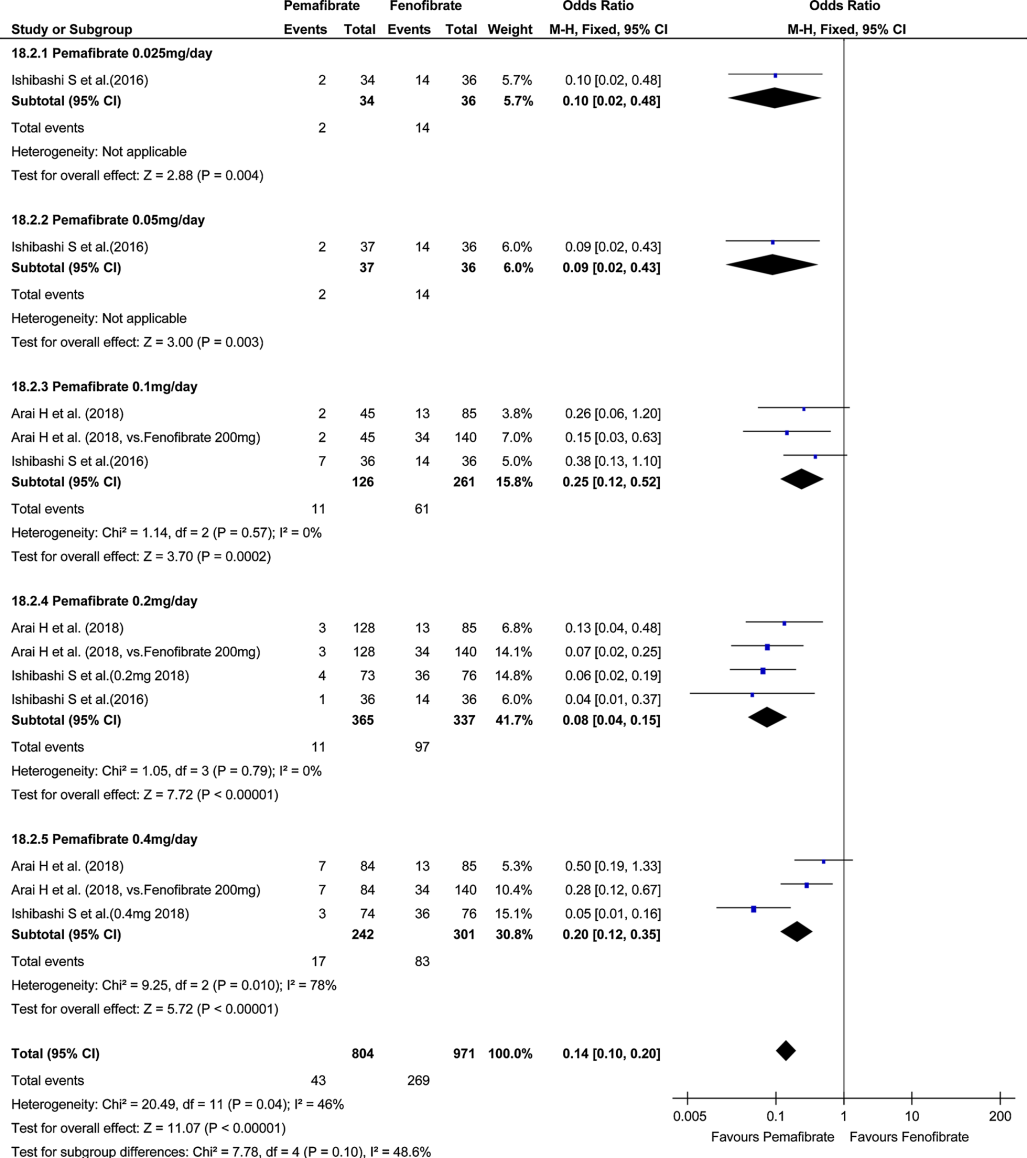
Forest plot presenting meta-analysis on OR for effect of pemafibrate versus fenofibrate on hepatobiliary enzyme. OR in the individual studies are presented as squares with 95% confidence intervals (CIs) presented as extending lines. The pooled OR with its 95% CI is depicted as a diamond. OR, odds ratio

Investigation of total AEs indicated no significant difference in total AEs between the pemafibrate group and the placebo group (OR, 0.92; 95% CI, 0.74–1.14; P = 0.45; Fig. 4). On the other hand, total AEs in the pemafibrate group was significantly lower than in the fenofibrate group (OR, 0.60; 95% CI, 0.49–0.73; P < 0.001; Fig. 5). The frequency of hepatobiliary enzyme activity increase was significantly lower in the pemafibrate group as compared with either the placebo group (OR, 0.33; 95% CI, 0.21–0.52; P < 0.001; Fig. 6) or the fenofibrate group (OR, 0.14; 95% CI, 0.10–0.20; P < 0.001; Fig. 7). In particular, ALT activity decreased significantly following administration of pemafibrate as compared with the placebo group while γGTP activity decreased significantly as compared with both the placebo group and the fenofibrate group.

Investigation of kidney dysfunction indicated no difference in the frequency of kidney dysfunction when comparing the pemafibrate group and the placebo group (OR, 1.67; 95% CI, 0.63–4.42; P = 0.30) (see Additional file 1: Fig. S9), or when comparing with the fenofibrate group (OR, 0.51; 95% CI, 0.20–1.32; P = 0.19) (see Additional file 1: Fig. S10). However, although Cre level increased in the pemafibrate group compared with the placebo group, the increase in Cre level in the pemafibrate group was significantly lower than in the fenofibrate group. No significant difference was found in the frequency of CK activity increase between the pemafibrate group and the placebo group (OR, 0.50; 95% CI, 0.24–1.05; P = 0.07) (see Additional file 1: Fig. S11), nor between the pemafibrate and fenofibrate groups (OR, 0.55; 95% CI, 0.21–1.45; P = 0.23) (see Additional file 1: Fig. S12).

## Discussion

Here, using a meta-analysis of RCTs, we investigated the efficacy and safety of pemafibrate administration in patients with dyslipidemia. With respect to efficacy, the TG, HDL-C, and non-HDL-C levels as well as HOMA-IR improved in the pemafibrate group compared with the placebo group. In contrast, the LDL-C level was higher in the pemafibrate group than in the placebo and fenofibrate groups. With respect to safety, the total AEs were not significantly different between the pemafibrate and placebo groups; however, an improvement in the hepatobiliary enzyme activity was observed in the pemafibrate group compared with the placebo group. Furthermore, compared with the fenofibrate group, the pemafibrate group showed an elevated hepatobiliary enzyme activity, the total AEs were significantly fewer, and the increase in Cre level was significantly lower.

When PPARα is activated in the liver, the expression of apolipoprotein C-III, which inhibits the activity of the TG-hydrolyzing lipoprotein lipase (LPL) enzyme, decreases, thereby causing increased LPL activity that leads to decreased blood TG levels [22]. Furthermore, the activation of PPARα induces the expression of apolipoprotein A-I and A-II, which are constituents of HDL-C, such that the HDL-C level increases [23]. In the present study, compared with the placebo group, although the pemafibrate group showed a decrease in the TG level and an increase in the HDL-C level, there was no significant difference with the levels in the fenofibrate group. However, with respect to safety, the frequency of hepatobiliary enzyme activity increase was lower in the pemafibrate group than in the placebo group and fenofibrate groups; indeed, an improvement in hepatobiliary enzyme activity was observed in the pemafibrate group. The activation of PPARα by pemafibrate reportedly accelerates fatty acid oxidation, which in turn promotes energy combustion in the liver and reduces fat accumulation [22, 23].

In the present study, ALT and γGTP activities were lower in the pemafibrate group than in the placebo group and γGTP activity decreased compared with the fenofibrate group; this ALT-improving effect was particularly marked in the groups administered the highest doses (0.2 and 0.4 mg/day) of pemafibrate, suggesting the likelihood of a dose-dependent effect. In animal experiments, fatty liver improvement, liver fibrosis suppression, and nonalcoholic steatohepatitis improvement have been reported following the administration of fibrate-type drugs [24, 25]. Considering it is difficult to continue the administration of fibrate-type drugs because of hepatic disorder development [8], pemafibrate may likely be useful from the viewpoint of its beneficial effects on the liver and its tolerability by the patient.

Although there were no differences in the HbA1c levels in the pemafibrate group compared with the placebo or fenofibrate groups, HOMA-IR decreased in the pemafibrate group compared with the placebo group. Activation of PPARα may lead to decreases in the hepatic fat mass owing to the promotion of β-oxidation in the liver, promotion of LPL production, and promotion of apolipoprotein A-I and A-II production [22, 23]. Increases in the hepatic fat mass, decreases in the TG levels, and increases in the HDL-C levels are associated with decreases in HOMA-IR [26]. Although the effects of PPARα agonists (other than pemafibrate) on HOMA-IR have been reported in humans, the consistency of the results is low [27–29]. In contrast, the results of the present study corroborate with those reported by previous studies on improved insulin resistance following pemafibrate administration [20, 30]. How insulin resistance improvement in response to pemafibrate administration influences sugar metabolism, lipid metabolism, and cardiovascular disease onset needs to be investigated in future studies.

In the present study, the LDL-C levels were higher in the pemafibrate group than in the placebo and fenofibrate groups. As mentioned earlier, the activation of PPARα increases the LPL levels and decreases the TG levels [22]. As with other fibrate-type drugs, the LDL-C levels assumedly increase as a result of very low-density lipoproteins containing large amounts of apolipoprotein B being converted to LDL-C owing to the catabolic action of pemafibrate [31, 32]. However, a previous study reported that LDL-C closely related to arteriosclerotic disease onset was markedly reduced by pemafibrate administration, whereas the increased LDL-C type ranged from medium to large LDL-C [11]. Therefore, although the increase in the LDL-C levels following pemafibrate administration is expected to be safe, further future investigations are required to elucidate the clinical significance of this effect. In contrast, with respect to the effects of pemafibrate administration on the Cre level, which was lower in the pemafibrate group than in the in the fenofibrate group, there was a slight increase compared with the placebo group. Many fibrate-type drugs, including fenofibrate, are renal excretory and may cause renal disorders [9]. In contrast, because pemafibrate is biliary excretory, it can hardly cause kidney damage [33]; this difference in pharmacokinetics has been inferred to be the cause of the results of the present study. Compared with placebo, although pemafibrate increases the Cre level to a very small extent, it seems necessary to carefully consider the potential risks as well as benefits of pemafibrate.

Pemafibrate has been developed with an aim of reducing TG, which is reported as a risk factor for development of cardiovascular diseases in patients with dyslipidemia or insulin resistance, or increasing HDL-C levels [10]. The present study demonstrated that the lipid-improving effect of pemafibrate is comparable with that of fenofibrate. Furthermore, pemafibrate showed fewer increases in total AEs and a smaller increase in hepatobiliary system enzyme activities than that showed by fenofibrate; these effects assumedly contribute to the high tolerability of pemafibrate by patients. PPARα assumedly has multifaceted effects, such as anti-inflammatory and antioxidant activities, in addition to its effect on lipids and has been proposed to suppress arteriosclerotic disease onset [34, 35]. A recent study has suggested the involvement of angiopoietin-like protein 8 (ANGPTL8) in the association between dyslipidemia and arteriosclerosis [36], indicating that hypertriglyceridemia is partially involved in the association between ANGPTL8 and arteriosclerosis [36]. A study has reported that ANGPTL8 may be involved in HDL-C dysfunction [37], and another study has indicated the close association of ANGPTL8 with dyslipidemia regardless of glucose intolerance or diabetes mellitus [38]. Taken together, these reports suggest that lipid management, including TG and HDL-C, is extremely important in the prevention of cardiovascular diseases. A large-scale clinical trial [entitled “Pemafibrate to Reduce Cardiovascular Outcomes by Reducing Triglycerides in Patients with Diabetes (PROMINENT)”] assessing the effect of pemafibrate administration on cardiovascular outcomes is underway [39], and the results of this trial are eagerly awaited.

The present study has several limitations. First, the RCTs included in this study involved only data from clinical trials. In the future, research data, including clinical research data, may have to be re-examined. Second, given the small number of RCTs included in the current meta-analysis, it is possible that some relevant data may not have been searched this time, thereby influencing the results and causing biases. Third, this study only included Japanese subjects; therefore, the results of this study cannot be generalized to other populations. Finally, the observation period of the RCTs included in this study was comparatively short and the results cannot be related to long-term efficacy and safety. Therefore, the results of the present study should be interpreted with caution.

## Conclusions

In summary, using a meta-analysis of RCTs, we investigated the efficacy and safety of pemafibrate administration in patients with dyslipidemia. The lipid profile significantly improved in the pemafibrate group than in the placebo group. Apart from the fact that the pemafibrate group showed a lipid-improving effect equivalent to that in the fenofibrate group, the total AEs was significantly fewer in the pemafibrate group than in the fenofibrate group and the hepatobiliary enzyme activity was actually improved. However, the clinical data of daily medical practice and the long-term efficacy and safety of pemafibrate administration need to be investigated in future studies.

## Additional file

**Additional file 1.** Additional figures and tables.

## Abbreviations

CI: confidence interval
RCTs: randomized controlled trials
SMD: standardized mean difference
OR: odds ratio
TG: triglycerides
HDL-C: high-density lipoprotein cholesterol
LDL-C: low-density lipoprotein cholesterol
Non-HDL-C: non-high-density lipoprotein cholesterol
HbA1c: hemoglobin A1c
HOMA-IR: homeostasis model assessment for insulin resistance
ALT: alanine aminotransferase
ALT: alanine aminotransferase
γGTP: γ-glutamyl transpeptidase
AEs: adverse events
PPARα: peroxisome proliferator-activated receptor α
SPPARMα: selective peroxisome proliferator-activated receptor α
CK: creatine kinase
LPL: lipoprotein lipase
