# Supplementary material for: Efficacy and safety of pemafibrate administration in patients with dyslipidemia: a systematic review and meta-analysis
Source: Cardiovasc Diabetol. 2019 Mar 21;18:38. doi: 10.1186/s12933-019-0845-x (PMC6429757; doi:10.1186/s12933-019-0845-x)
Supplement: Supplementary file 1 — Additional file 1. Additional figures and tables. [file 12933_2019_845_MOESM1_ESM.docx]

**ADDITIONAL FILE 1**

**Additional tables and figures.**

**Table S1. Risk of bias assessment included in the meta-analysis**

| **No** | **Reference** | **Randomization procedure** | **Allocation concealment** | **Blinding of personnel and participants** | **Blinding of outcome assessment** | **Incomplete outcome assessment** | **Selective reporting** |
| --- | --- | --- | --- | --- | --- | --- | --- |
| 1 | [11] | L | L | L | L | L | L |
| 2 | [17] | U | U | U | U | L | L |
| 3 | [12] | L | L | L | L | L | L |
| 4 | [18] | L | L | L | L | L | L |
| 5 | [19] | L | L | L | L | L | L |
| 6 | [20] | L | L | L | L | H | L |
| 7 | [21] | L | L | L | L | L | L |

**Abbreviations: L, low risk of bias; U, unclear risk of bias; H, high risk of bias**

**
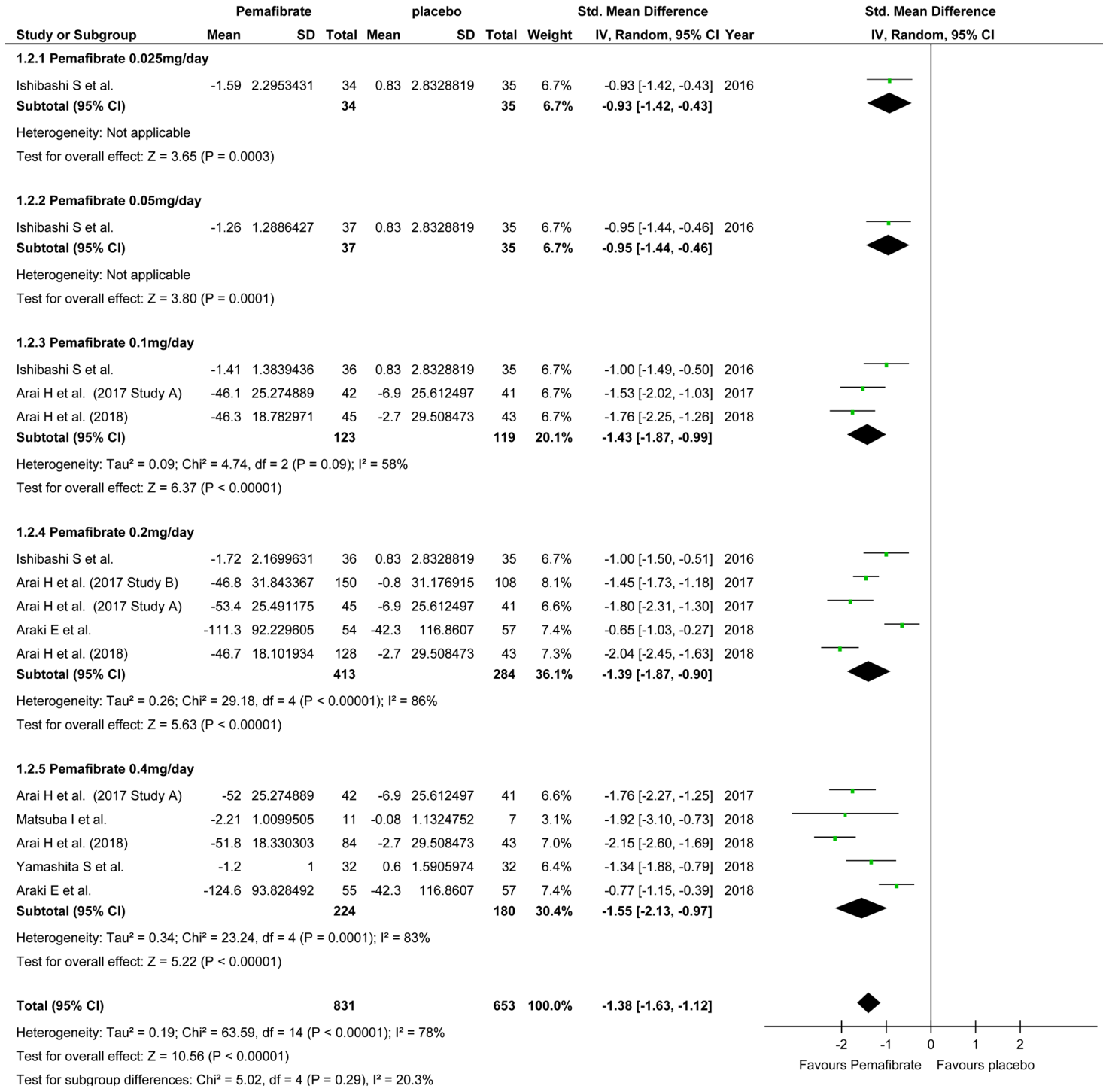
**

**Fig S1. Forest plot presenting the meta-analysis based on standardized mean differences (SMDs) for the effect of pemafibrate versus placebo on HDL-C.**

SMDs in the individual studies are presented as squares with 95% confidence intervals (CIs) presented as extending lines. The pooled SMD with its 95% CI is depicted as a diamond.

HDL-C, high-density lipoprotein-cholesterol

**
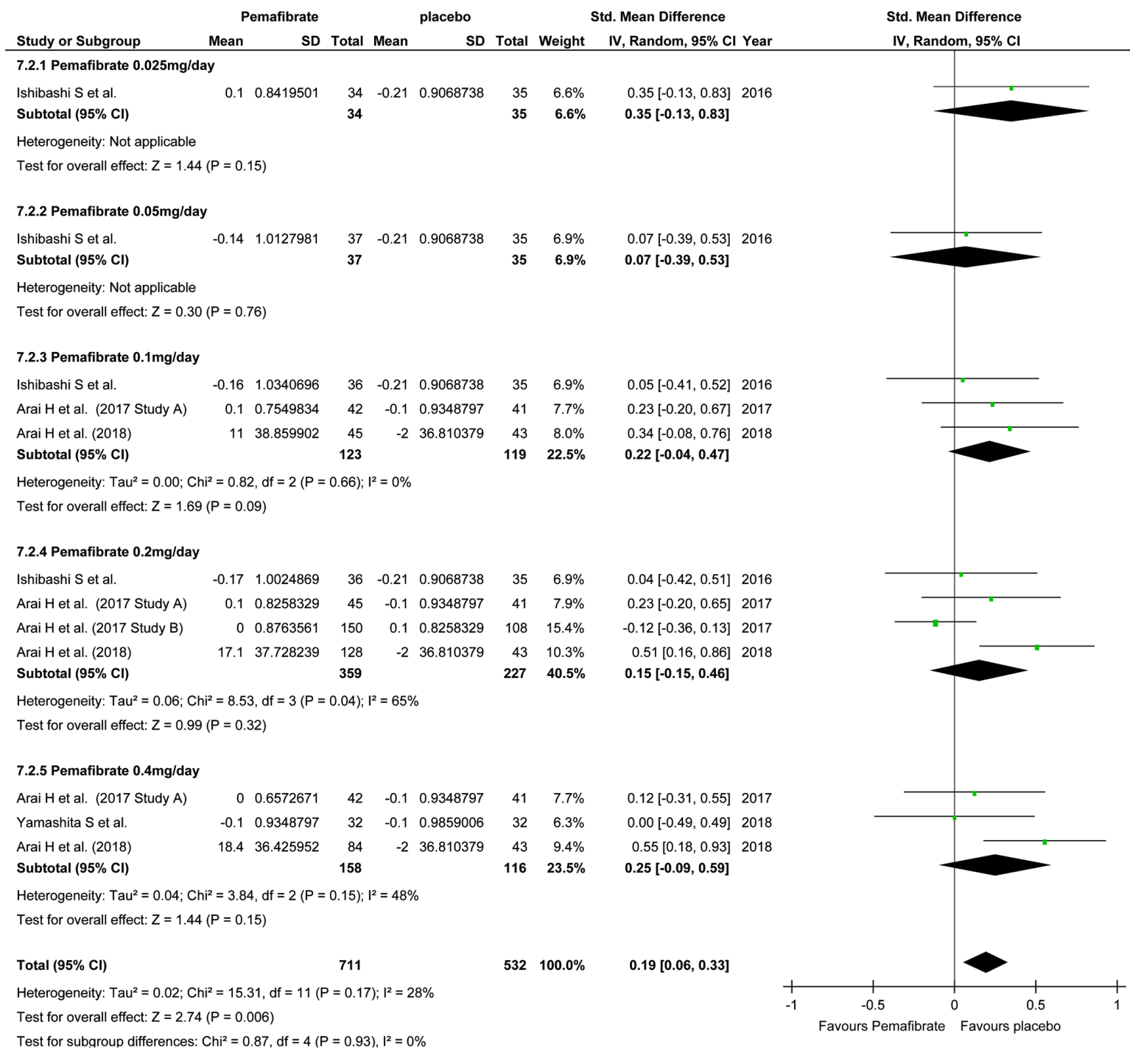
**

**Fig S2. Forest plot presenting the meta-analysis based on standardized mean differences (SMDs) for the effect of pemafibrate versus placebo on LDL-C.**

SMDs in the individual studies are presented as squares with 95% confidence intervals (CIs) presented as extending lines. The pooled SMD with its 95% CI is depicted as a diamond.

LDL-C, low-density lipoprotein-cholesterol

**
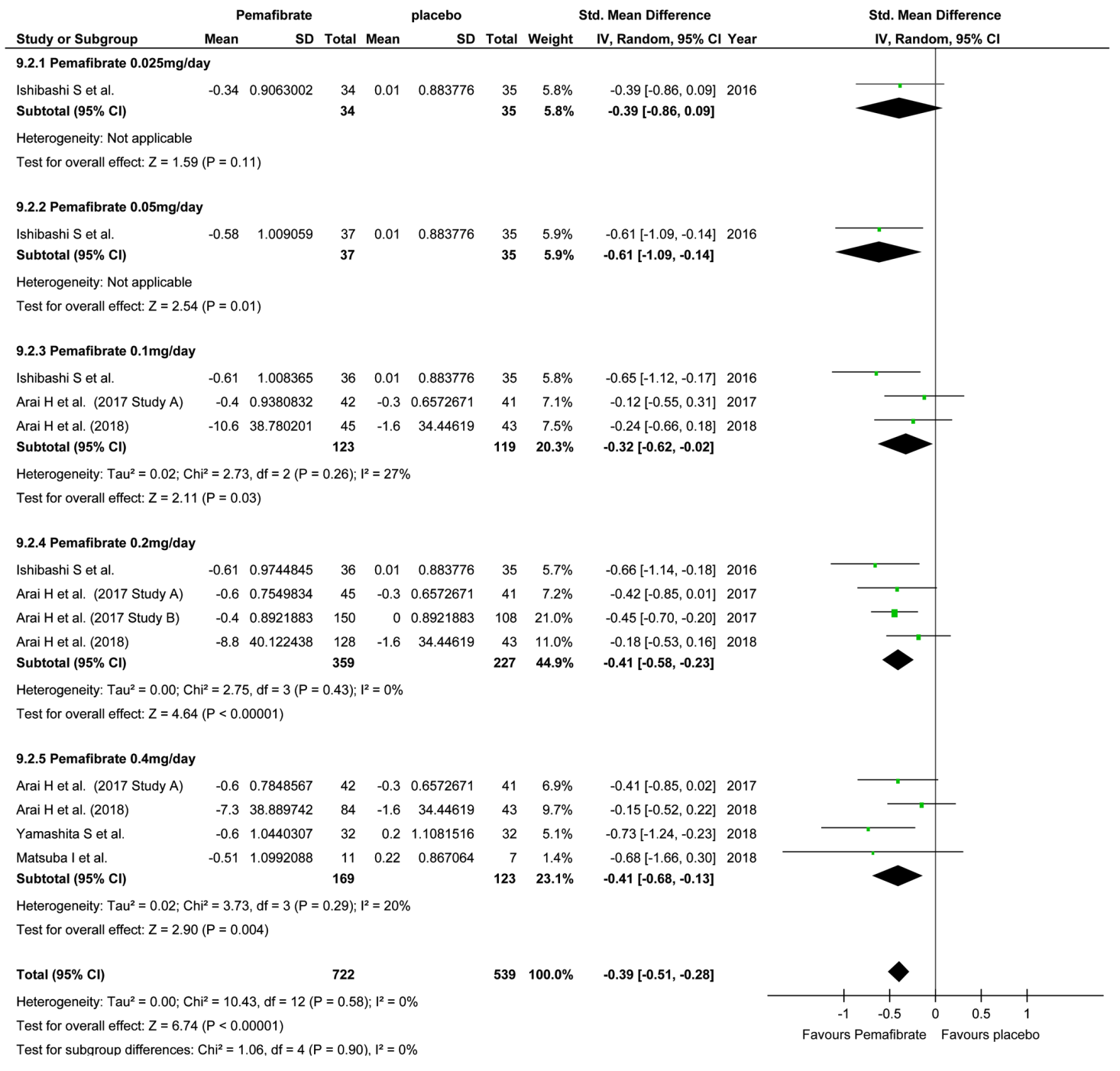
**

**Fig S3. Forest plot presenting the meta-analysis based on standardized mean differences (SMDs) for the effect of pemafibrate versus placebo on Non-HDL-C.** SMDs in the individual studies are presented as squares with 95% confidence intervals (CIs) presented as extending lines. The pooled SMD with its 95% CI is depicted as a diamond.

Non-HDL-C, high-density lipoprotein-cholesterol

**
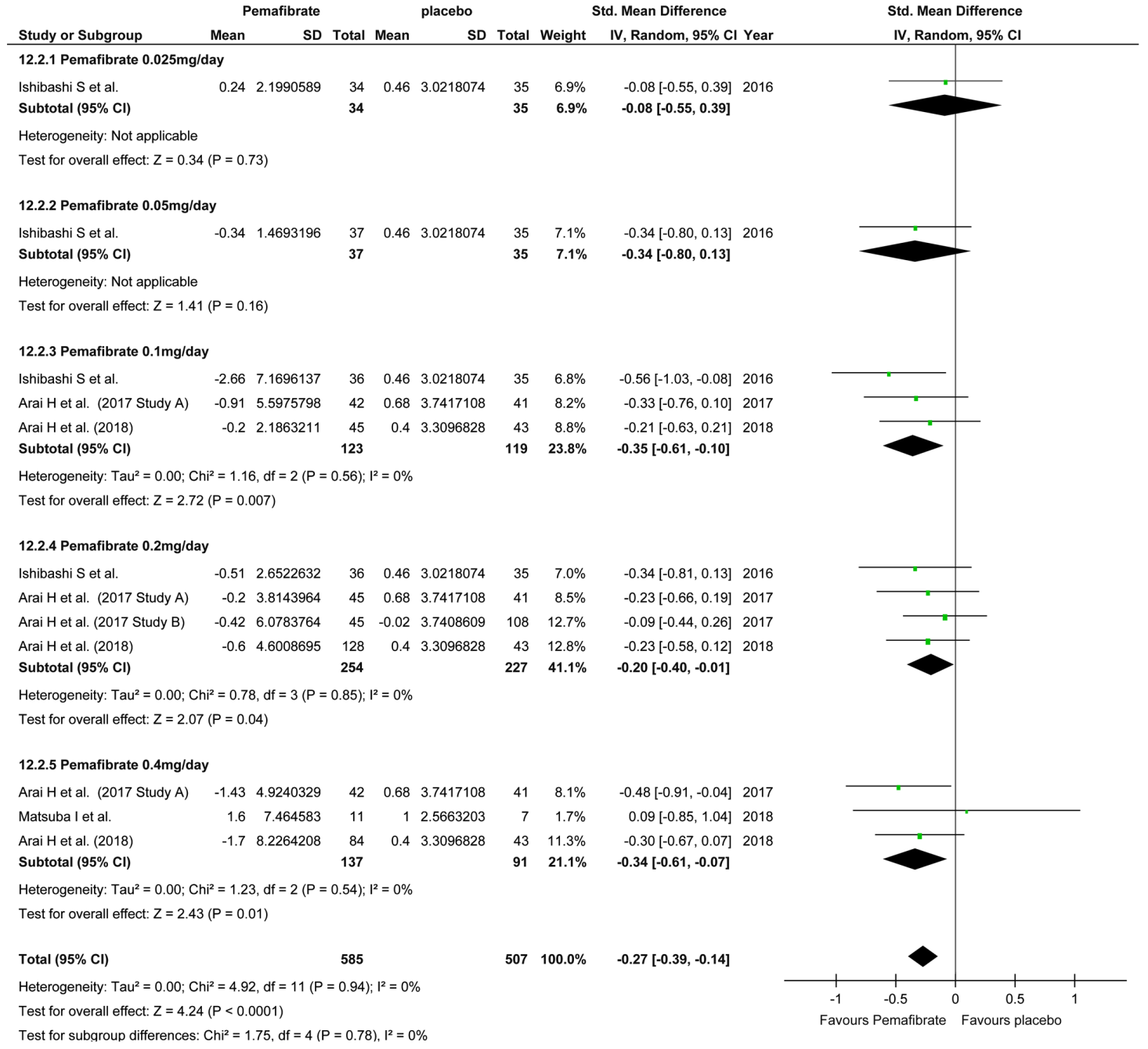
**

**Fig S4. Forest plot presenting the meta-analysis based on standardized mean differences (SMDs) for the effect of pemafibrate versus placebo on HOMA-IR.** SMDs in the individual studies are presented as squares with 95% confidence intervals (CIs) presented as extending lines. The pooled SMD with its 95% CI is depicted as a diamond.

HOMA-IR, Homeostasis model assessment for insulin resistance

**
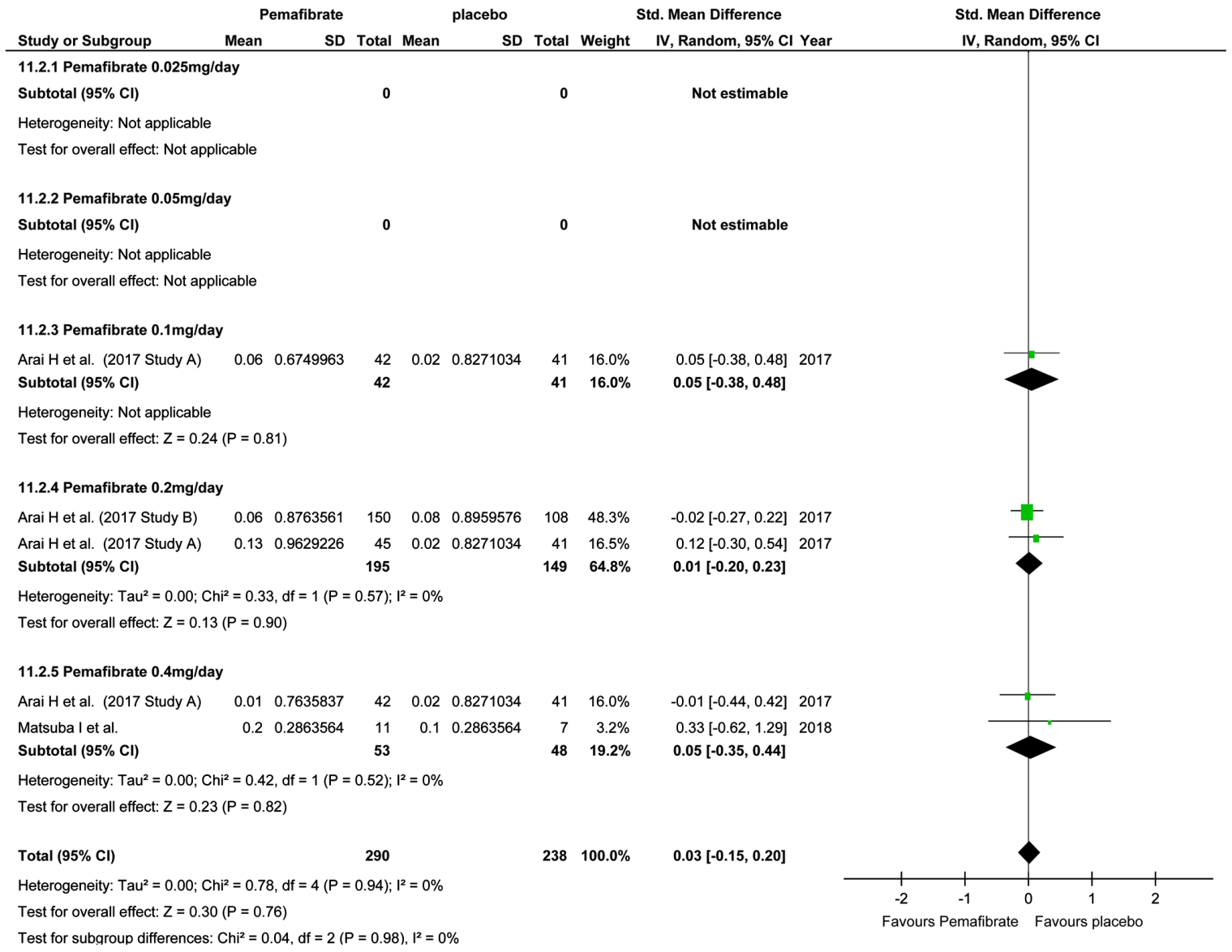
**

**Fig S5. Forest plot presenting the meta-analysis based on standardized mean differences (SMDs) for the effect of pemafibrate versus placebo on HbA1c.**

SMDs in the individual studies are presented as squares with 95% confidence intervals (CIs) presented as extending lines. The pooled SMD with its 95% CI is depicted as a diamond.

HbA1c, Hemoglobin A1c

**
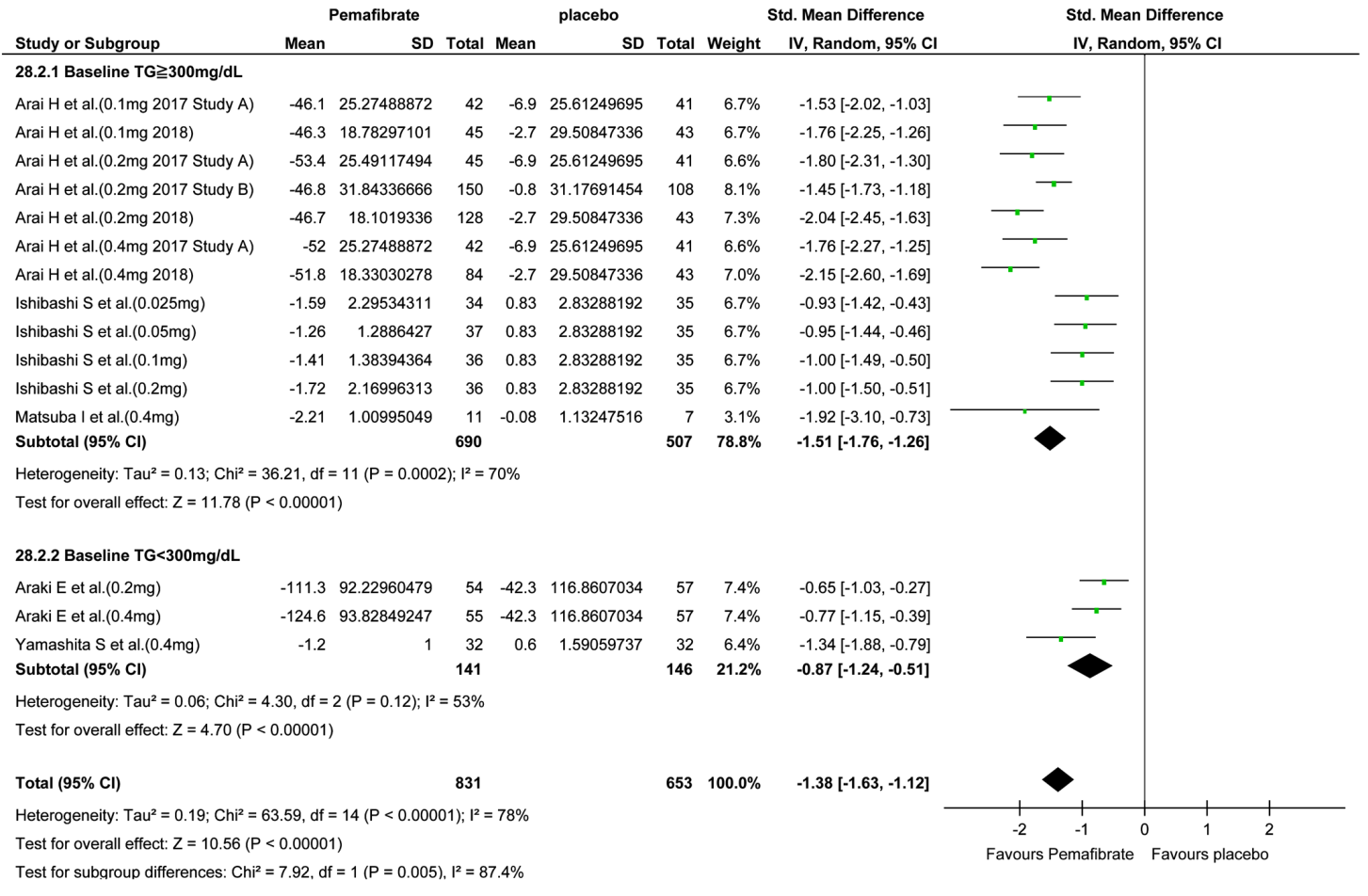
**

**Fig S6. Subgroup analysis according to baseline TG category. Forest plot presenting the meta-analysis based on standardized mean differences (SMDs) for the effect of pemafibrate versus placebo on TG.**

SMDs in the individual studies are presented as squares with 95% confidence intervals (CIs) presented as extending lines. The pooled SMD with its 95% CI is depicted as a diamond.

**
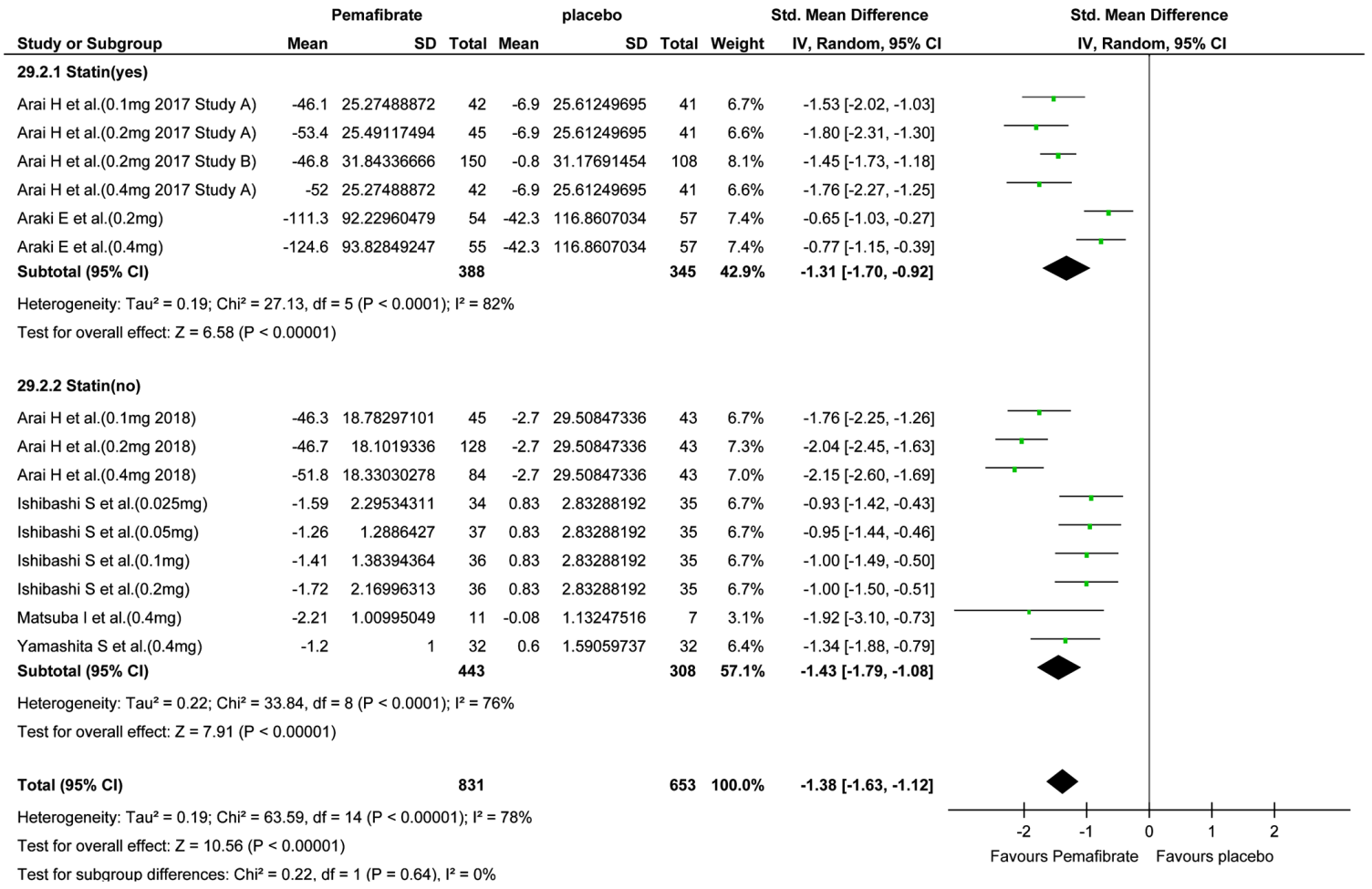
**

**Fig S7. Subgroup analysis according to statin combination or non- statin combination category.**

Forest plot presenting the meta-analysis based on standardized mean differences (SMDs) for the effect of pemafibrate versus placebo on TG. SMDs in the individual studies are presented as squares with 95% confidence intervals (CIs) presented as extending lines. The pooled SMD with its 95% CI is depicted as a diamond.

**
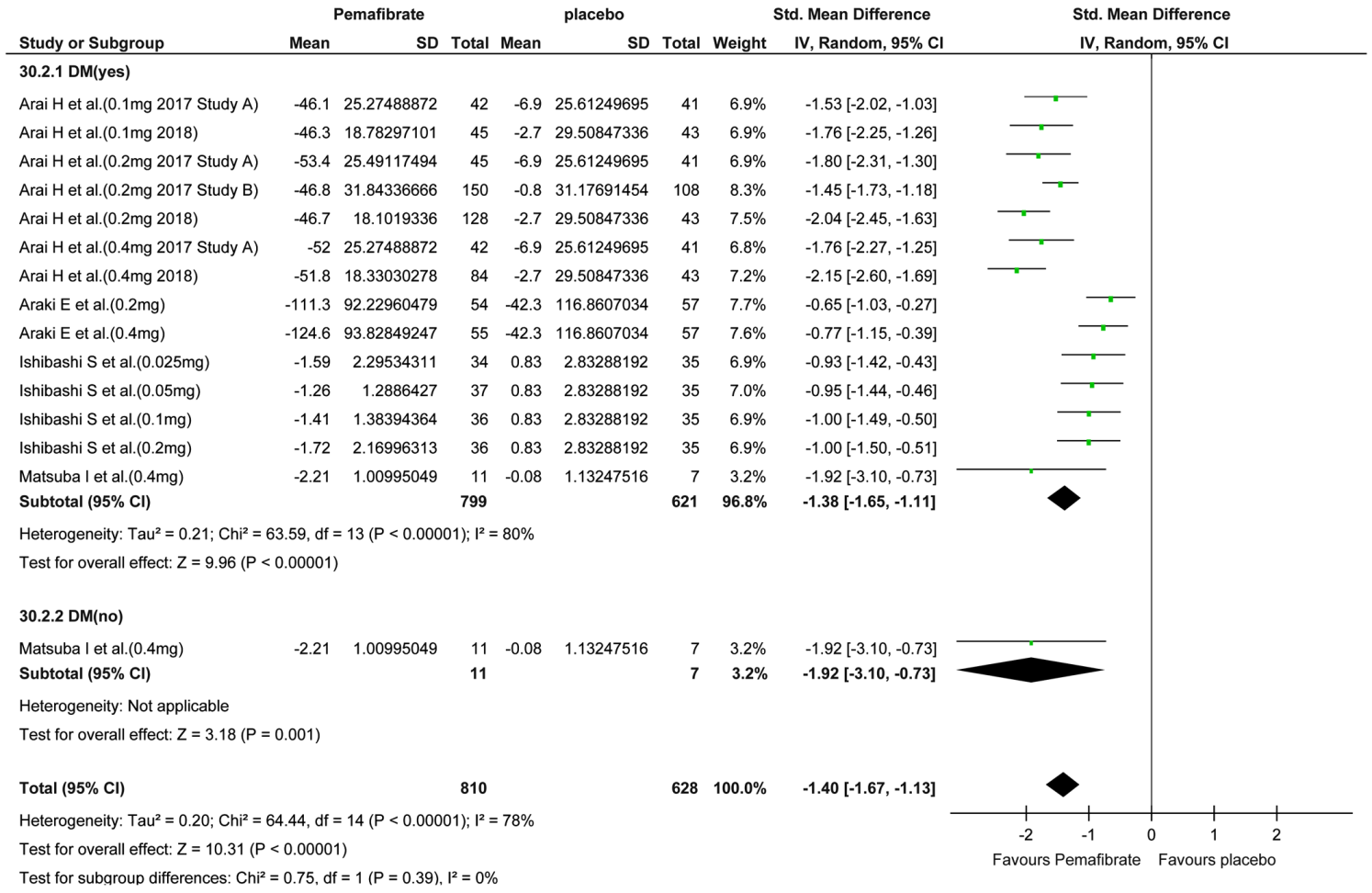
**

**Fig S8. Subgroup analysis according to diabetes or non-diabetes category. Forest plot presenting the meta-analysis based on standardized mean differences (SMDs) for the effect of pemafibrate versus placebo on TG.**

SMDs in the individual studies are presented as squares with 95% confidence intervals (CIs) presented as extending lines. The pooled SMD with its 95% CI is depicted as a diamond.

**
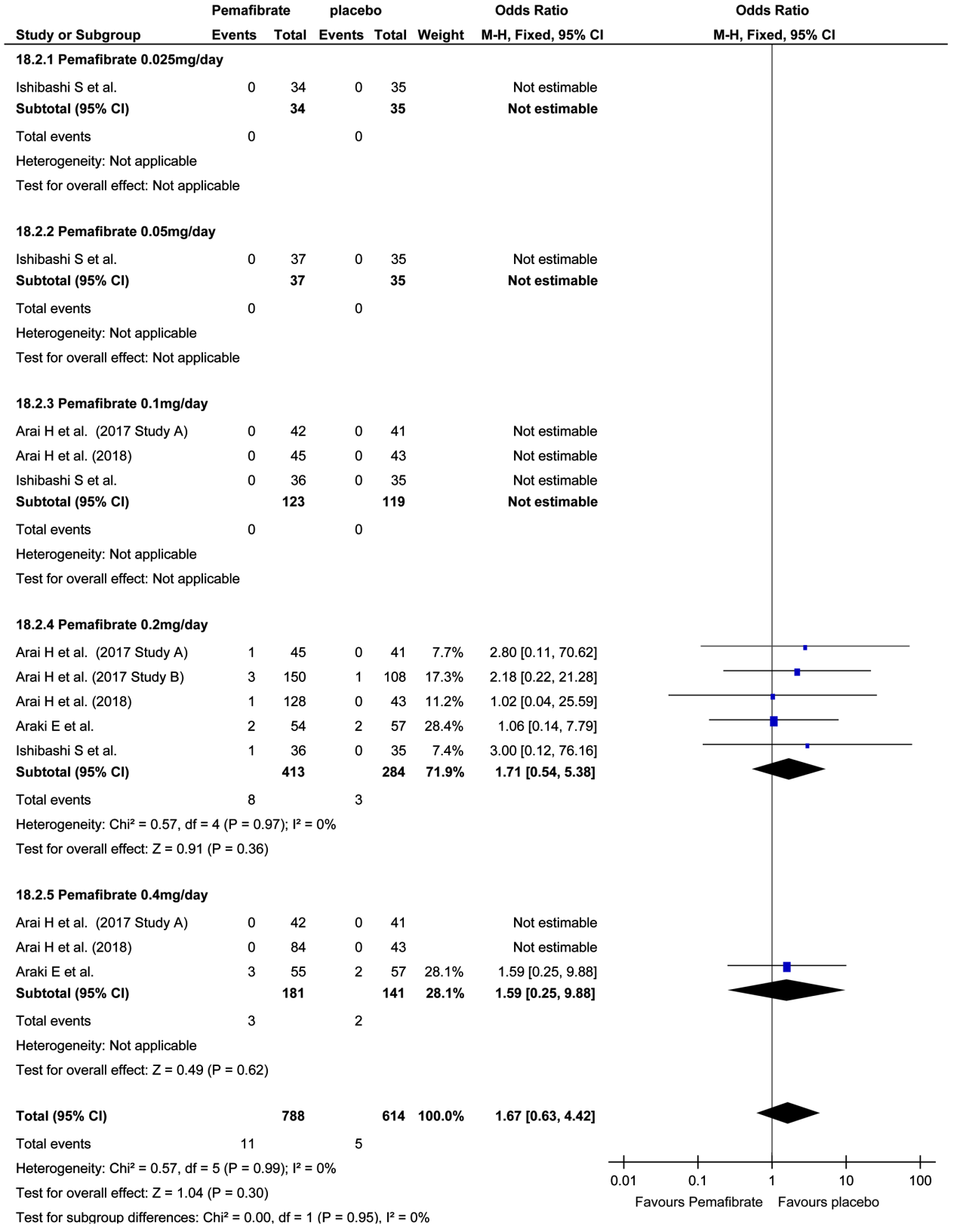
**

**Fig S9. Forest plot presenting the meta-analysis based on odds ratio (OR) for the effect of pemafibrate versus placebo on creatinine increased.**

OR in the individual studies are presented as squares with 95% confidence intervals (CIs) presented as extending lines. The pooled OR with its 95% CI is depicted as a diamond.

**
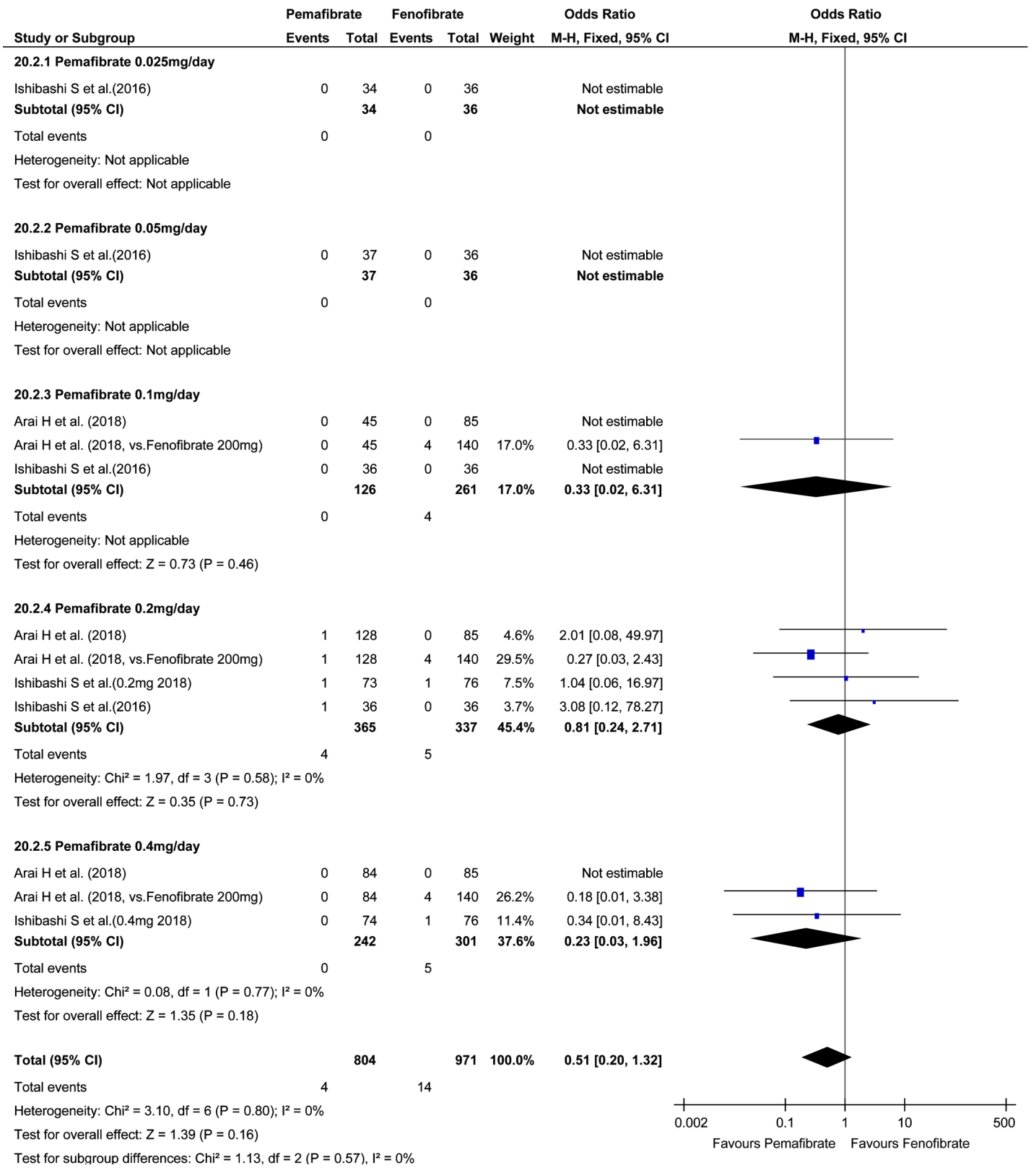
**

**Fig S10. Forest plot presenting the meta-analysis based on odds ratio (OR) for the effect of pemafibrate versus fenofibrate on creatinine increased.**

OR in the individual studies are presented as squares with 95% confidence intervals (CIs) presented as extending lines. The pooled OR with its 95% CI is depicted as a diamond.

**
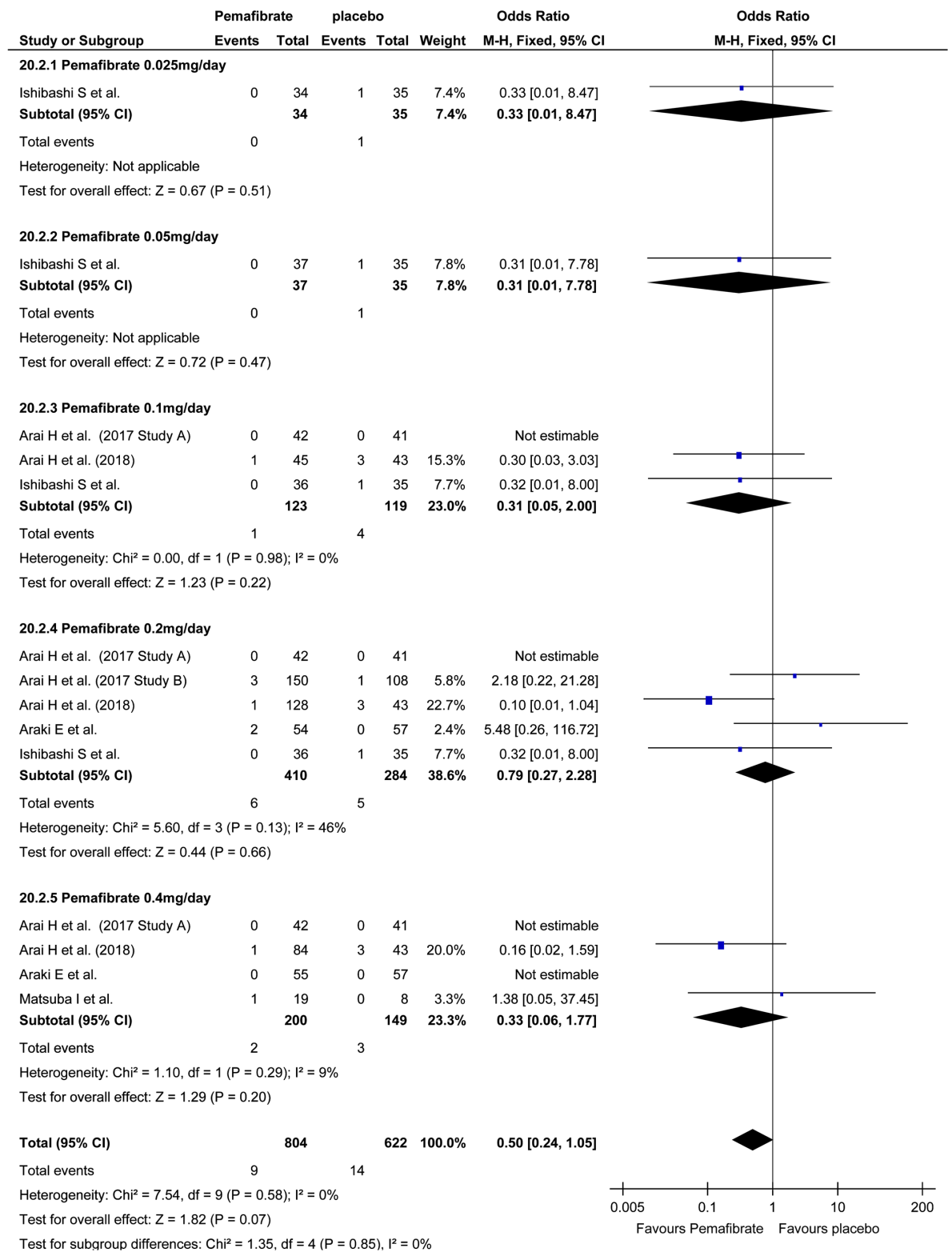
**

**Fig S11. Forest plot presenting the meta-analysis based on odds ratio (OR) for the effect of pemafibrate versus placebo on CK increased.**

OR in the individual studies are presented as squares with 95% confidence intervals (CIs) presented as extending lines. The pooled OR with its 95% CI is depicted as a diamond.

CK, creatine kinase

**
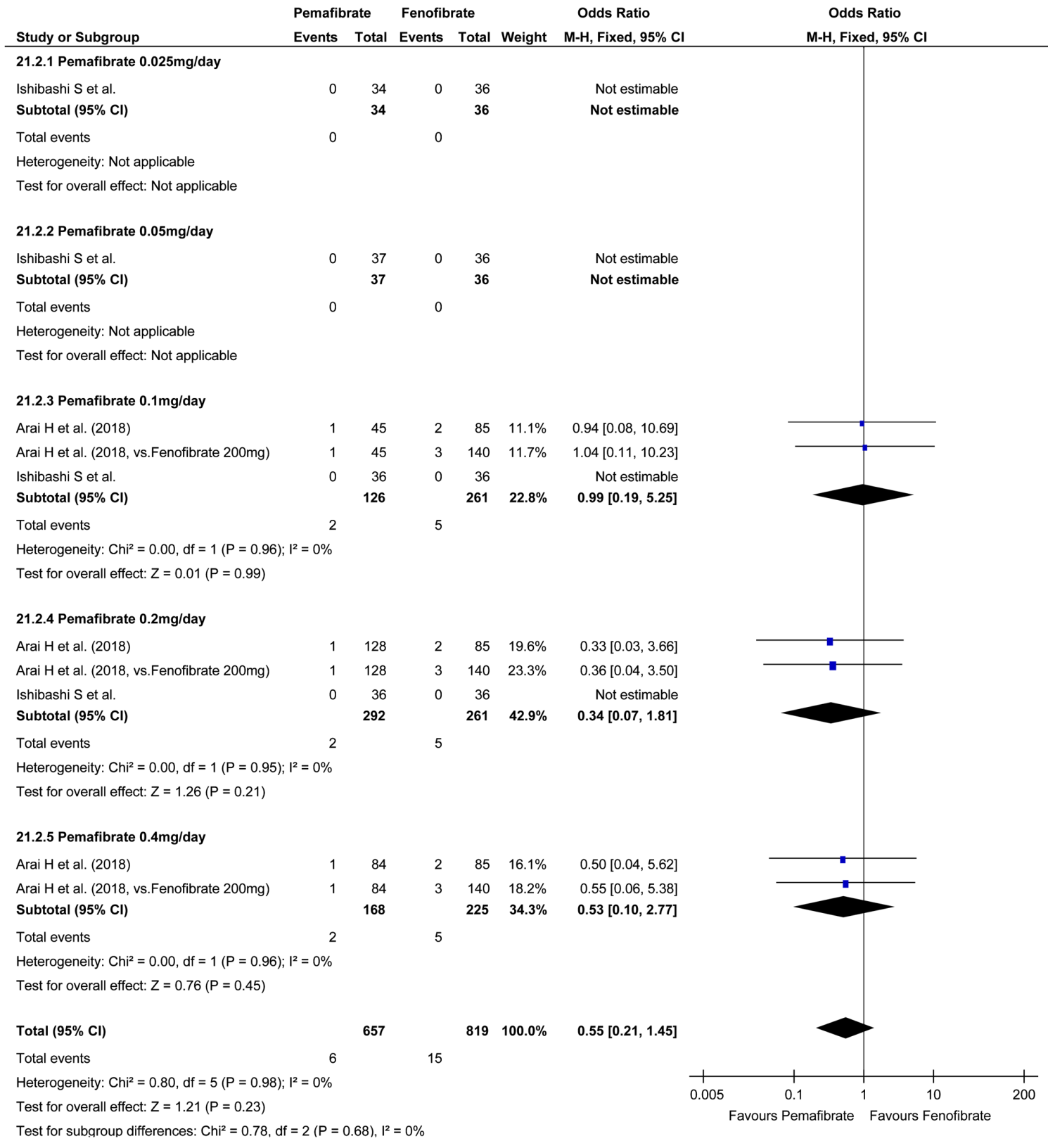
**

**Fig S12. Forest plot presenting the meta-analysis based on odds ratio (OR) for the effect of pemafibrate versus fenofibrate on CK increased.**

OR in the individual studies are presented as squares with 95% confidence intervals (CIs) presented as extending lines. The pooled OR with its 95% CI is depicted as a diamond.

CK, creatine kinase
